# 2,4,5-Trimeth­oxy­benzaldehyde monohydrate

**DOI:** 10.1107/S160053681004794X

**Published:** 2010-11-24

**Authors:** Abdullah M. Asiri, Salman A. Khan, M. Nawaz Tahir

**Affiliations:** a The Center of Excellence for Advanced Materials Research, King Abdul Aziz University, Jeddah 21589, PO Box 80203, Saudi Arabia; b Department of Chemistry, Faculty of Science, King Abdul Aziz University, Jeddah 21589, PO Box 80203, Saudi Arabia; c Department of Physics, University of Sargodha, Sargodha, Pakistan

## Abstract

In the title compound, C_10_H_12_O_4_·H_2_O, the 2,4,5-trimeth­oxy­benzaldehyde mol­ecule is almost planar (rms deviation = 0.0183 Å). There is an *R*_1_^2^(5) ring motif due to O—H⋯O hydrogen bonding. In the crystal, the mol­ecules are stabilized in the form of one-dimensional polymeric chains extending along [010] due to O—H⋯O hydrogen bonding with adjacent water mol­ecules. The H atoms involved in inter­molecular hydrogen bonding are disordered over two sets of sites of equal occupancy.

## Related literature

For related background and related structures, see: Asiri *et al.* (2010**a* ▶,b* ▶), Hussain *et al.* (2010 ▶). For graph-set notation, see: Bernstein *et al.* (1995 ▶).
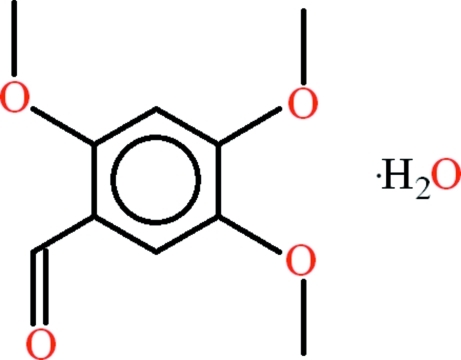


## Experimental

### 

#### Crystal data

C_10_H_12_O_4_·H_2_O
*M* *_r_* = 214.21
Monoclinic, 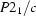

*a* = 18.084 (5) Å
*b* = 4.2456 (10) Å
*c* = 14.600 (4) Å
β = 108.290 (9)°
*V* = 1064.3 (5) Å^3^
*Z* = 4
Mo *K*α radiation
μ = 0.11 mm^−1^
*T* = 296 K
0.22 × 0.10 × 0.08 mm

#### Data collection

Bruker Kappa APEXII CCD diffractometer
Absorption correction: multi-scan (*SADABS*; Bruker, 2005 ▶) *T*_min_ = 0.992, *T*_max_ = 0.995
8287 measured reflections
1915 independent reflections
983 reflections with *I* > 2σ(*I*)
*R*_int_ = 0.066

#### Refinement

*R*[*F* ^2^ > 2σ(*F* ^2^)] = 0.060
*wR*(*F* ^2^) = 0.212
*S* = 1.05
1915 reflections
148 parameters
3 restraints
H atoms treated by a mixture of independent and constrained refinement
Δρ_max_ = 0.18 e Å^−3^
Δρ_min_ = −0.22 e Å^−3^

### 

Data collection: *APEX2* (Bruker, 2009 ▶); cell refinement: *SAINT* (Bruker, 2009 ▶); data reduction: *SAINT*; program(s) used to solve structure: *SHELXS97* (Sheldrick, 2008 ▶); program(s) used to refine structure: *SHELXL97* (Sheldrick, 2008 ▶); molecular graphics: *ORTEP-3 for Windows* (Farrugia, 1997 ▶) and *Mercury* (Bruno *et al.*, 2002 ▶); software used to prepare material for publication: *WinGX* (Farrugia, 1999 ▶) and *PLATON* (Spek, 2009 ▶).

## Supplementary Material

Crystal structure: contains datablocks global, I. DOI: 10.1107/S160053681004794X/dn2621sup1.cif

Structure factors: contains datablocks I. DOI: 10.1107/S160053681004794X/dn2621Isup2.hkl

Additional supplementary materials:  crystallographic information; 3D view; checkCIF report

## Figures and Tables

**Table 1:** Hydrogen-bond geometry (Å, °)

*D*—H⋯*A*	*D*—H	H⋯*A*	*D*⋯*A*	*D*—H⋯*A*
O5—H51⋯O2	0.85 (4)	2.54 (5)	3.181 (5)	133 (4)
O5—H51⋯O3	0.85 (4)	2.19 (4)	3.006 (5)	160 (4)
O5—H52⋯O5^i^	0.83 (10)	1.89 (10)	2.710 (6)	174 (19)
O5—H53⋯O5^ii^	0.86 (10)	1.86 (10)	2.714 (6)	169 (7)

Symmetry codes: (i) 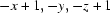; (ii) 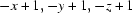.

**Fig. 1.**
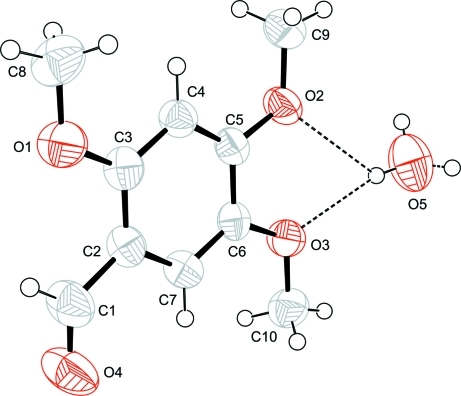
View of the title compound with the atom numbering scheme. The thermal ellipsoids are drawn at the 50% probability level. H-atoms are shown as small spheres of arbitrary radii. The dotted lines represent the intramolecular H-bondings or bonds of disordered H-atoms.

**Fig. 2.**
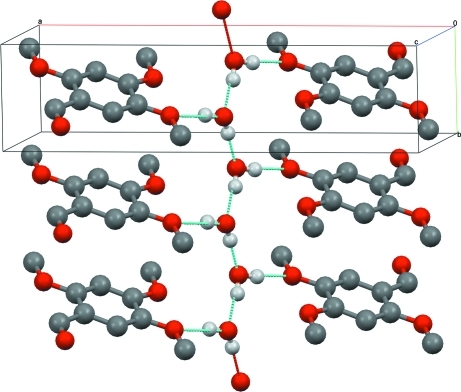
Partial packing view showing the polymeric chains extending along the b axis.

## supplementary crystallographic information

### Comment

The crystal structure of (II) *i.e.*, (*E*)-1-(2,5-dimethyl-3-thienyl)-3- (2,4,5-trimethoxyphenyl)prop-2-en-1-one (Asiri *et al.*, 2010*a*), (III) *i.e.*, 3,4-dimethyl-*N*-(2,4,5-trimethoxybenzylidene)-1,2-isoxazol-5-amine (Asiri *et al.*, 2010*b*) and (IV) *i.e.*, 2,3-Dimethyl-N-[(E)-2,4,5 -trimethoxybenzylidene]aniline (Hussain *et al.*, 2010) have been published which contain the aldehyde moiety. The title compound (I, Fig. 1) is being reported here in which the aldehyde has reacted with water instead of aniline.

In (I), the 2,4,5-trimethoxybenzaldehyde is planar with r. m. s. deviation of 0.0183 Å. One of the H-atoms of water molecule is disordered over two set of sites with equal occupancy ratio. The non-disordered H-atom of H_2_O makes H-bonding with adjacent two methoxy groups through O—H···O type and complete an *R*_1_^2^(5) ring motif (Bernstein *et al.*, 1995). The disordered H-atoms of H_2_O makes H-bonding of O—H···O type with adjacent water molecules (Table 1, Fig. 2). Due to these H-bondings molecules are stabilized in the form of one dimensional polymeric chains extending along the *b* axis i.e [010]. There does not appear any appreciable π interaction.

### Experimental

A mixture of 2,4,5-methoxy benzaldehyde (0.50 g, 2.5 mmol) and 4*H*-[1,2,4] Triazol-3-ylamine (0.21 g, 2.5 mmol) in ethanol (15 ml) was refluxed for 5 h with stirring to give a light yellow precipitate. This material was filtered off and washed with ethanol to give the starting aldehyde coordinated to water. m.p. 376 K

### Refinement

The coordinates of H-atoms of water molecule are refined under distance restraints. One H-atom of H_2_O is disordered over two sites with equal occupancy ratio. The H-atoms were positioned geometrically (C–H = 0.93–0.96 Å) and refined as riding with *U*_iso_(H) = *xU*_eq_(C, O), where *x* = 1.5 for methyl and *x* = 1.2 for other H-atoms.

### Crystal data

**Table:**

C_10_H_12_O_4_·H_2_O	*F*(000) = 456
*M**_r_* = 214.21	*D*_x_ = 1.337 Mg m^−^^3^
Monoclinic, *P*2_1_/*c*	Mo *K*α radiation, λ = 0.71073 Å
Hall symbol: -P 2ybc	Cell parameters from 983 reflections
*a* = 18.084 (5) Å	θ = 2.4–25.3°
*b* = 4.2456 (10) Å	µ = 0.11 mm^−^^1^
*c* = 14.600 (4) Å	*T* = 296 K
β = 108.290 (9)°	Needle, colourless
*V* = 1064.3 (5) Å^3^	0.22 × 0.10 × 0.08 mm
*Z* = 4	

### Data collection

**Table:**

Bruker Kappa APEXII CCD diffractometer	1915 independent reflections
Radiation source: fine-focus sealed tube	983 reflections with *I* > 2σ(*I*)
graphite	*R*_int_ = 0.066
Detector resolution: 8.20 pixels mm^-1^	θ_max_ = 25.3°, θ_min_ = 2.4°
ω scans	*h* = −21→21
Absorption correction: multi-scan (*SADABS*; Bruker, 2005)	*k* = −3→5
*T*_min_ = 0.992, *T*_max_ = 0.995	*l* = −17→17
8287 measured reflections	

### Refinement

**Table:**

Refinement on *F*^2^	Primary atom site location: structure-invariant direct methods
Least-squares matrix: full	Secondary atom site location: difference Fourier map
*R*[*F*^2^ > 2σ(*F*^2^)] = 0.060	Hydrogen site location: inferred from neighbouring sites
*wR*(*F*^2^) = 0.212	H atoms treated by a mixture of independent and constrained refinement
*S* = 1.05	*w* = 1/[σ^2^(*F*_o_^2^) + (0.0973*P*)^2^ + 0.249*P*] where *P* = (*F*_o_^2^ + 2*F*_c_^2^)/3
1915 reflections	(Δ/σ)_max_ < 0.001
148 parameters	Δρ_max_ = 0.18 e Å^−^^3^
3 restraints	Δρ_min_ = −0.22 e Å^−^^3^

### Special details

**Table:**

Geometry. Bond distances, angles *etc*. have been calculated using the rounded fractional coordinates. All su's are estimated from the variances of the (full) variance-covariance matrix. The cell e.s.d.'s are taken into account in the estimation of distances, angles and torsion angles
Refinement. Refinement of *F*^2^ against ALL reflections. The weighted *R*-factor *wR* and goodness of fit *S* are based on *F*^2^, conventional *R*-factors *R* are based on *F*, with *F* set to zero for negative *F*^2^. The threshold expression of *F*^2^ > σ(*F*^2^) is used only for calculating *R*-factors(gt) *etc*. and is not relevant to the choice of reflections for refinement. *R*-factors based on *F*^2^ are statistically about twice as large as those based on *F*, and *R*- factors based on ALL data will be even larger.

### Fractional atomic coordinates and isotropic or equivalent isotropic displacement parameters (Å^2^)

**Table:**

	*x*	*y*	*z*	*U*_iso_*/*U*_eq_	Occ. (<1)
O1	0.08087 (15)	0.7631 (7)	0.1135 (2)	0.0705 (11)	
O2	0.33240 (14)	0.6231 (6)	0.35470 (16)	0.0572 (9)	
O3	0.38101 (14)	0.2960 (6)	0.23792 (17)	0.0588 (9)	
O4	0.14718 (16)	0.2497 (8)	−0.0759 (2)	0.0873 (14)	
C1	0.1290 (2)	0.4010 (10)	−0.0160 (3)	0.0697 (16)	
C2	0.1803 (2)	0.4721 (8)	0.0809 (2)	0.0501 (12)	
C3	0.1560 (2)	0.6509 (8)	0.1459 (3)	0.0511 (12)	
C4	0.2054 (2)	0.7063 (8)	0.2389 (3)	0.0480 (12)	
C5	0.2800 (2)	0.5845 (7)	0.2667 (2)	0.0452 (11)	
C6	0.3059 (2)	0.4044 (8)	0.2018 (2)	0.0454 (12)	
C7	0.2562 (2)	0.3514 (8)	0.1105 (3)	0.0479 (12)	
C8	0.0523 (2)	0.9331 (10)	0.1787 (3)	0.0740 (16)	
C9	0.3107 (2)	0.8019 (9)	0.4243 (3)	0.0635 (16)	
C10	0.4117 (2)	0.1230 (9)	0.1747 (3)	0.0636 (14)	
O5	0.4918 (2)	0.2497 (10)	0.4398 (3)	0.0985 (18)	
H1	0.07829	0.47778	−0.03301	0.0837*	
H4	0.18830	0.82431	0.28205	0.0578*	
H7	0.27321	0.23288	0.06748	0.0575*	
H8A	0.08262	1.12102	0.19862	0.1111*	
H8B	0.05607	0.80482	0.23412	0.1111*	
H8C	−0.00117	0.98883	0.14757	0.1111*	
H9A	0.26526	0.71063	0.43414	0.0950*	
H9B	0.29959	1.01451	0.40177	0.0950*	
H9C	0.35269	0.80171	0.48406	0.0950*	
H10A	0.41096	0.25222	0.12043	0.0949*	
H10B	0.38052	−0.06151	0.15245	0.0949*	
H10C	0.46429	0.06141	0.20837	0.0949*	
H51	0.452 (2)	0.274 (13)	0.390 (3)	0.1180*	
H52	0.498 (9)	0.09 (2)	0.474 (9)	0.1180*	0.500
H53	0.492 (9)	0.42 (2)	0.472 (9)	0.1180*	0.500

### Atomic displacement parameters (Å^2^)

**Table:**

	*U*^11^	*U*^22^	*U*^33^	*U*^12^	*U*^13^	*U*^23^
O1	0.0409 (16)	0.094 (2)	0.0703 (19)	0.0102 (14)	0.0084 (14)	−0.0162 (15)
O2	0.0539 (16)	0.0718 (16)	0.0397 (14)	0.0065 (12)	0.0060 (13)	−0.0050 (12)
O3	0.0494 (16)	0.0691 (17)	0.0519 (16)	0.0141 (13)	0.0071 (13)	−0.0061 (12)
O4	0.056 (2)	0.140 (3)	0.0593 (19)	0.0016 (17)	0.0087 (16)	−0.0300 (19)
C1	0.047 (2)	0.095 (3)	0.059 (3)	0.000 (2)	0.005 (2)	−0.012 (2)
C2	0.043 (2)	0.058 (2)	0.048 (2)	−0.0036 (18)	0.0126 (18)	0.0013 (18)
C3	0.038 (2)	0.058 (2)	0.056 (2)	−0.0028 (17)	0.0127 (19)	0.0030 (18)
C4	0.045 (2)	0.053 (2)	0.045 (2)	0.0003 (17)	0.0127 (18)	−0.0022 (16)
C5	0.047 (2)	0.0455 (19)	0.040 (2)	−0.0056 (17)	0.0092 (18)	0.0024 (15)
C6	0.040 (2)	0.050 (2)	0.045 (2)	0.0006 (16)	0.0115 (18)	0.0065 (16)
C7	0.044 (2)	0.055 (2)	0.047 (2)	−0.0051 (17)	0.0177 (18)	−0.0022 (17)
C8	0.045 (2)	0.080 (3)	0.095 (3)	0.005 (2)	0.019 (2)	−0.011 (2)
C9	0.068 (3)	0.068 (3)	0.050 (2)	0.000 (2)	0.012 (2)	−0.0073 (19)
C10	0.052 (2)	0.067 (2)	0.069 (3)	0.0106 (19)	0.015 (2)	−0.005 (2)
O5	0.074 (2)	0.124 (4)	0.083 (3)	0.002 (3)	0.004 (2)	0.020 (2)

### Geometric parameters (Å, °)

**Table:**

O1—C3	1.376 (5)	C5—C6	1.407 (5)
O1—C8	1.415 (5)	C6—C7	1.372 (5)
O2—C5	1.346 (4)	C1—H1	0.9300
O2—C9	1.419 (5)	C4—H4	0.9300
O3—C6	1.373 (4)	C7—H7	0.9300
O3—C10	1.422 (5)	C8—H8A	0.9600
O4—C1	1.211 (5)	C8—H8B	0.9600
O5—H53	0.86 (10)	C8—H8C	0.9600
O5—H51	0.85 (4)	C9—H9C	0.9600
O5—H52	0.83 (10)	C9—H9A	0.9600
C1—C2	1.459 (5)	C9—H9B	0.9600
C2—C3	1.390 (5)	C10—H10A	0.9600
C2—C7	1.400 (5)	C10—H10B	0.9600
C3—C4	1.391 (6)	C10—H10C	0.9600
C4—C5	1.382 (5)		
			
O2···O3	2.559 (4)	H4···C9	2.5200
O2···O5	3.181 (5)	H4···H9B	2.3600
O3···O5	3.006 (5)	H4···H9A	2.2700
O3···O2	2.559 (4)	H4···C8	2.4900
O5···O5^i^	2.714 (6)	H7···O4	2.5700
O5···C10^ii^	3.191 (6)	H7···C10	2.5500
O5···O3	3.006 (5)	H7···H10A	2.3700
O5···O5^iii^	2.710 (6)	H7···H10B	2.3100
O5···O2	3.181 (5)	H8A···C3^vii^	2.8400
O1···H1	2.4500	H8A···C4	2.7500
O2···H51	2.54 (5)	H8A···H4	2.2900
O2···H9B^iv^	2.7900	H8B···C4	2.7100
O3···H10C^ii^	2.8900	H8B···C8^xii^	3.0800
O3···H51	2.19 (4)	H8B···H4	2.2700
O4···H9A^v^	2.8600	H8C···O4^vi^	2.7100
O4···H7	2.5700	H8C···C1^vi^	3.0000
O4···H8C^vi^	2.7100	H9A···H4	2.2700
O5···H9C^i^	2.6900	H9A···C4	2.7100
O5···H10C^ii^	2.8500	H9A···O4^xiii^	2.8600
O5···H52^iii^	1.89 (10)	H9B···C4	2.7800
O5···H53^i^	1.86 (11)	H9B···O2^vii^	2.7900
C4···C7^vii^	3.597 (5)	H9B···H4	2.3600
C7···C4^iv^	3.597 (5)	H9B···C5^vii^	3.0700
C7···C9^viii^	3.494 (6)	H9C···O5^i^	2.6900
C9···C7^ix^	3.494 (6)	H9C···H10B^xiii^	2.5600
C10···O5^x^	3.191 (6)	H10A···H7	2.3700
C1···H8C^vi^	3.0000	H10A···C7	2.7900
C3···H8A^iv^	2.8400	H10B···C6^iv^	2.8400
C4···H8B	2.7100	H10B···H9C^v^	2.5600
C4···H9A	2.7100	H10B···H7	2.3100
C4···H9B	2.7800	H10B···C7	2.7600
C4···H8A	2.7500	H10C···O5^x^	2.8500
C5···H9B^iv^	3.0700	H10C···O3^x^	2.8900
C6···H10B^vii^	2.8400	H10C···H10C^ii^	2.5800
C7···H10B	2.7600	H10C···C10^x^	3.0000
C7···H10A	2.7900	H10C···H10C^x^	2.5800
C8···H8B^xi^	3.0800	H51···O2	2.54 (5)
C8···H4	2.4900	H51···O3	2.19 (4)
C9···H4	2.5200	H51···C10	3.06 (4)
C10···H7	2.5500	H51···H52^iii^	2.45 (12)
C10···H10C^ii^	3.0000	H51···H53^i^	2.34 (13)
C10···H51	3.06 (4)	H52···O5^iii^	1.89 (10)
H1···O1	2.4500	H52···H51^iii^	2.45 (12)
H4···H8A	2.2900	H53···O5^i^	1.86 (10)
H4···H8B	2.2700	H53···H51^i^	2.34 (12)
			
C3—O1—C8	118.4 (3)	C3—C4—H4	120.00
C5—O2—C9	118.6 (3)	C5—C4—H4	120.00
C6—O3—C10	117.7 (3)	C6—C7—H7	119.00
H52—O5—H53	112 (10)	C2—C7—H7	119.00
H51—O5—H52	122 (10)	O1—C8—H8A	109.00
H51—O5—H53	103 (10)	O1—C8—H8B	109.00
O4—C1—C2	125.2 (4)	O1—C8—H8C	109.00
C3—C2—C7	118.6 (3)	H8B—C8—H8C	109.00
C1—C2—C3	122.3 (3)	H8A—C8—H8B	109.00
C1—C2—C7	119.1 (3)	H8A—C8—H8C	109.00
O1—C3—C2	116.4 (3)	O2—C9—H9B	109.00
O1—C3—C4	122.6 (3)	H9A—C9—H9C	110.00
C2—C3—C4	121.0 (3)	O2—C9—H9C	109.00
C3—C4—C5	119.5 (3)	H9A—C9—H9B	110.00
C4—C5—C6	120.4 (3)	O2—C9—H9A	109.00
O2—C5—C6	115.2 (3)	H9B—C9—H9C	109.00
O2—C5—C4	124.4 (3)	O3—C10—H10C	110.00
C5—C6—C7	119.2 (3)	H10A—C10—H10C	109.00
O3—C6—C7	125.8 (3)	H10B—C10—H10C	109.00
O3—C6—C5	114.9 (3)	H10A—C10—H10B	109.00
C2—C7—C6	121.3 (3)	O3—C10—H10A	109.00
O4—C1—H1	117.00	O3—C10—H10B	109.00
C2—C1—H1	117.00		
			
C8—O1—C3—C2	176.9 (3)	C1—C2—C7—C6	−178.2 (3)
C8—O1—C3—C4	−2.0 (5)	C3—C2—C7—C6	0.6 (5)
C9—O2—C5—C4	−0.4 (5)	O1—C3—C4—C5	179.6 (3)
C9—O2—C5—C6	−180.0 (3)	C2—C3—C4—C5	0.7 (5)
C10—O3—C6—C5	−177.6 (3)	C3—C4—C5—O2	−179.9 (3)
C10—O3—C6—C7	2.5 (5)	C3—C4—C5—C6	−0.3 (5)
O4—C1—C2—C3	179.2 (4)	O2—C5—C6—O3	−0.3 (4)
O4—C1—C2—C7	−2.1 (6)	O2—C5—C6—C7	179.7 (3)
C1—C2—C3—O1	−1.1 (5)	C4—C5—C6—O3	−179.9 (3)
C1—C2—C3—C4	177.9 (3)	C4—C5—C6—C7	0.1 (5)
C7—C2—C3—O1	−179.8 (3)	O3—C6—C7—C2	179.7 (3)
C7—C2—C3—C4	−0.8 (5)	C5—C6—C7—C2	−0.2 (5)

Symmetry codes: (i) −*x*+1, −*y*+1, −*z*+1; (ii) −*x*+1, *y*+1/2, −*z*+1/2; (iii) −*x*+1, −*y*, −*z*+1; (iv) *x*, *y*−1, *z*; (v) *x*, −*y*+1/2, *z*−1/2; (vi) −*x*, −*y*+1, −*z*; (vii) *x*, *y*+1, *z*; (viii) *x*, −*y*+3/2, *z*−1/2; (ix) *x*, −*y*+3/2, *z*+1/2; (x) −*x*+1, *y*−1/2, −*z*+1/2; (xi) −*x*, *y*+1/2, −*z*+1/2; (xii) −*x*, *y*−1/2, −*z*+1/2; (xiii) *x*, −*y*+1/2, *z*+1/2.

### Hydrogen-bond geometry (Å, °)

**Table:**

*D*—H···*A*	*D*—H	H···*A*	*D*···*A*	*D*—H···*A*
O5—H51···O2	0.85 (4)	2.54 (5)	3.181 (5)	133 (4)
O5—H51···O3	0.85 (4)	2.19 (4)	3.006 (5)	160 (4)
O5—H52···O5^iii^	0.83 (10)	1.89 (10)	2.710 (6)	174 (19)
O5—H53···O5^i^	0.86 (10)	1.86 (10)	2.714 (6)	169 (7)

Symmetry codes: (iii) −*x*+1, −*y*, −*z*+1; (i) −*x*+1, −*y*+1, −*z*+1.
